# The Impact of Bevacizumab and miR200c on EMT and EGFR-TKI Resistance in EGFR-Mutant Lung Cancer Organoids

**DOI:** 10.3390/genes15121624

**Published:** 2024-12-19

**Authors:** Nobuaki Kobayashi, Seigo Katakura, Nobuhiko Fukuda, Kohei Somekawa, Ayami Kaneko, Takeshi Kaneko

**Affiliations:** 1 Department of Pulmonology, Yokohama City University Graduate School of Medicine, 3-9 Fukuura, Yokohama 236-0004, Japan; 2 Department of Thoracic Oncology, Kanagawa Cancer Center, 2-3-2 Nakao, Yokohama 241-0815, Japan

**Keywords:** non-small-cell lung cancer (NSCLC), *Epidermal Growth Factor Receptor* (*EGFR*) mutations, epithelial–mesenchymal transition (EMT), drug resistance, organoid models

## Abstract

**Objectives**: This research aims to investigate the mechanisms of resistance to epidermal growth factor receptor tyrosine kinase inhibitors (EGFR-TKIs) in non-small-cell lung cancer (NSCLC), particularly focusing on the role of the epithelial–mesenchymal transition (EMT) within the tumor microenvironment (TME). **Materials and Methods**: We employed an in vitro three-dimensional organoid model that mirrors the physiology of human lung cancer. These organoids consist of lung cancer cells harboring specific *EGFR* mutations, human mesenchymal stem cells, and human umbilical vein endothelial cells. We analyzed EMT and drug resistance markers, and evaluated the effects of the anti-angiogenic agent Bevacizumab and micro-RNA miR200c. **Results**: The study identified a significant link between EMT and EGFR-TKI resistance. Notable findings included a decrease in E-cadherin and an increase in Zinc Finger E-Box Binding Homeobox 1 (ZEB1), both of which influenced EMT and resistance to treatment. Bevacizumab showed promise in improving drug resistance and mitigating EMT, suggesting an involvement of the Vascular Endothelial Growth Factor (VEGF) cascade. Transfection with miR200c was associated with improved EMT and drug resistance, further highlighting the role of EMT in TKI resistance. **Conclusions**: Our research provides significant insights into the EMT-driven EGFR-TKI resistance in NSCLC and offers potential strategies to overcome resistance, including the use of Bevacizumab and miR200c. However, due to the limitations in organoid models in replicating precise human cancer TME and the potential influence of specific *EGFR* mutations, further in vivo studies and clinical trials are necessary for validation.

## 1. Introduction

Non-small-cell lung cancer (NSCLC) remains the foremost cause of cancer-related deaths worldwide [1]. The identification of oncogenic driver mutations has transformed the therapeutic landscape for NSCLC, with *Epidermal Growth Factor Receptor* (*EGFR*) mutations observed in approximately 15–50% of advanced cases [2]. Predominantly comprising exon 19 deletions or L858R mutations in exon 21, these EGFR alterations serve as key targets for EGFR tyrosine kinase inhibitors (TKIs)—a therapeutic intervention that significantly enhances the prognosis of inoperable NSCLC patients [3,4].

Nevertheless, acquired resistance to EGFR-TKIs remains a considerable clinical challenge, typically emerging between 11 and 18 months following treatment initiation [5,6]. This resistance often develops through complex compensatory feedback mechanisms. One crucial pathway involves the upregulation of AXL, a receptor tyrosine kinase that promotes epithelial–mesenchymal transition (EMT) and drug resistance [7,8]. Additionally, EGFR inhibition can trigger the activation of other Human Epidermal growth factor Receptor (HER) family receptors, particularly HER2 and HER3, which restore downstream signaling pathways and contribute to treatment resistance [4,9]. Several mechanisms, including genetic mutations [10,11], histological transformation [12], and EMT [13], contribute to this resistance, with EMT being particularly instrumental in enabling cancer cell invasion and metastasis. The combination of EGFR-TKIs with Bevacizumab, an anti- Vascular Endothelial Growth Factor (VEGF) antibody, has emerged as a promising strategy to overcome these resistance mechanisms. Bevacizumab may counteract resistance by modulating the tumor microenvironment (TME), potentially affecting AXL signaling pathways and the mesenchymal state of cancer cells. This dual-targeting approach of EGFR-driven proliferation and VEGF-mediated angiogenesis represents a potentially powerful strategy to overcome drug resistance [14].

The complexity of these TME-dependent resistance mechanisms necessitates sophisticated in vitro models for their elucidation. Among these, organoids—self-organizing, multicellular structures capable of mimicking in vivo physiology and tissue-specific functions—hold substantial promise. Through the co-culture of lung cancer cells, human mesenchymal stem cells (MSCs), and human umbilical vein endothelial cells (HUVECs) on low-attachment plates, organoids that encapsulate the intricate cellular interactions integral to EMT can be generated [15,16,17].

In this study, we constructed an in vitro EMT model of EGFR-mutant lung cancer organoids to identify potential EMT-regulating compounds. We delved into the interplay between the TME and EGFR-TKI resistance, aiming to contribute to the broader understanding of EMT and its role in anticancer drug resistance.

## 2. Materials and Methods

### 2.1. Cell Lines

The luciferase-tagged human lung adenocarcinoma cell lines HCC827 and H1975 were procured from the Japanese Cancer Research Resources Bank. HCC827 cell lines exhibited Exon 19 deletions, while H1975 had L858R and T790M mutations on the *EGFR* gene. The cancer cells were maintained in Roswell Park Memorial Institute (RPMI) medium (Sigma-Aldrich, St. Louis, MO, USA), supplemented with 10% fetal bovine serum (FBS) (Biowest, Nuaillé, France) and 1% Pen/Strep (Gibco, Waltham, MA, USA) in a 5% CO_2_ environment at 37 °C. MSC and HUVEC were procured from TAKARA (Shiga, Japan). MSCs were cultivated in Mesenchymal Stem Cell Growth Medium 2 (PromoCell, Heidelberg, Germany) at 37 °C in 5% CO_2_. HUVECs were cultivated in Endothelial Cell Growth Medium 2 (EGM) (PromoCell) at 37 °C in 5% CO_2_. Both MSCs and HUVECs were utilized before their 10th passage.

### 2.2. The Construction of Human Lung Cancer Spheroids and Organoids

Matrigel (Corning, Corning, NY, USA) was combined in equal parts with RPMI medium. This mixture (30 µL) was seeded onto a cold 96-well plate (Corning), which had been cooled using phosphate-buffered saline (PBS) (Nihon Gene, Tokyo, Japan). Following an incubation period at 37 °C for 1 h, 1.11 × 10^5^ cancer cells were seeded to create spheroids. For organoids, 3.0 × 10^4^ cancer cells, 6.0 × 10^4^ MSCs, and 2.1 × 10^4^ HUVECs were seeded. The resultant cell pellets were resuspended in 30 µL drops on a 96 well plate. After a 30 min incubation at 37 °C, 200 µL of culture medium (a 1:1 mixture of RPMI medium and EGM) was added to each well upon complete gelation. Plates were then transferred to humidified 37 °C/5% CO_2_ incubators to allow 3D structures to form for subsequent analyses.

### 2.3. Detection of Epithelial–Mesenchymal Transition (EMT)

The expression of key EMT markers was assessed to evaluate the phenotypic changes associated with tumor progression and drug resistance. E-cadherin, a critical component of adherens junctions, was selected as a marker for epithelial differentiation. Loss of E-cadherin expression is a hallmark of EMT and is associated with increased cell motility and invasiveness. Conversely, zinc finger E-box-binding homeobox 1 (ZEB1), a transcriptional repressor of E-cadherin, was chosen as a marker of the mesenchymal phenotype. Upregulation of ZEB1 is known to drive EMT by directly suppressing E-cadherin expression and promoting the expression of other mesenchymal genes [18]. The expression of E-cadherin and ZEB1 was assessed using immunofluorescence staining and Western blotting.

### 2.4. Mimicking Micro-RNA200c (miR200c)

Lipofectamine RNAiMAX Reagent (Invitrogen, Carlsbad, CA, USA) and mimic miR200c (Eurofins Genomics, Tokyo, Japan) were directly added to the culture medium for organoid transfection. S-TuD-NC (Eurofins Genomics) was used as a negative control. Each well received 1 pmol of final mimic miR200c. The sequence was as follows: mimic miR200c (upstream primer, 5′-TAATACTGCCGGGTAATGATGGA-3′). The transfection incubation period was one day.

### 2.5. Total RNA Isolation, Reverse-Transcription and Polymerase Chain Reaction (PCR)

Total RNA was extracted from the organoids using the RNeasy mini kit (QIAGEN, Venlo, The Netherlands). The concentration of the extracted total RNA was determined using the Nanodrop 2000 system (Thermo Fisher Scientific, Waltham, MA, USA). The Mir-X miRNA First-Strand Synthesis Kit (TAKARA, Shiga, Japan) was used to convert RNAs into complementary DNA (cDNA). Reverse-transcription–quantitative Polymerase Chain Reaction (RT-qPCR) was conducted using the Mir-X miRNA qRT-PCR TB Green Kit (TAKARA), the reverse-transcription samples, and the miR200c primers (upstream primer, 5′-TAATACTGCCGGGTAATGATGGA-3′) (Eurofins Genomics) on the CFX96 Touch Real-Time PCR Detection System (Bio-Rad, Hercules, CA, USA). U6 primers (TAKARA) were used to normalize miRNA expression via the 2-ΔΔCq method (ΔCq = Cqtarget—Cqrereference).

### 2.6. Immunohistochemical Analyses

The spheroids and organoids were cultured for 72 h, fixed with 4% paraformaldehyde overnight, then paraffin-embedded and sectioned. After deparaffinization, morphology was examined via Hematoxylin and Eosin (HE) staining. These slices were stained with anti-EGFR antibody (Nichirei Biosciences, Tokyo, Japan) and anti-mouse IgG conjugated with horseradish peroxidase (HRP) (Dako, Carpinteria, CA, USA) for immunohistochemical staining.

### 2.7. Cell Viability

After 24 h from seeding, 1 nM–10 µM of EGFR-TKIs (Gefitinib, Med Chem Express, NJ, USA/Afatinib, Med Chem Express/Osimertinib, Med Chem Express) was added. The spheroids and organoids were co-cultured with EGFR-TKIs ± Bev for 72 h. An amount of 100 µL of the cell culture lysis reagent (Promega, Madison, WI, USA) was added and suspended. The mixture was frozen and thawed repeatedly. After 24 h, 20 µL of samples with 20 µL of luciferase substrate (Promega) was transferred onto the 96-well plate (Thermo Fisher Scientific, Waltham, MA, USA) and luciferase reporter activities were measured by a luminometer (PerkinElmer, MA, USA) to calculate the survival rate of cells. Luciferase activity was normalized using untreated controls for each condition, with the initial seeding of 1.11 × 10^5^ cancer cells for spheroids and 3.0 × 10^4^ cancer cells, 6.0 × 10^4^ MSCs, and 2.1 × 10^4^ HUVECs for organoids. After 72 h of culture, measurements were normalized to respective untreated controls and expressed as relative luciferase units (RLUs), with control values set at 100%. Measurements were performed under standardized conditions using consistent reagent volumes and luminometer settings. Averages of the half maximal inhibitory concentration (IC50) from at least three independent experiments were taken.

### 2.8. Immunofluorescence Analyses

The preparation of samples for immunofluorescence analyses paralleled the procedure used for immunohistochemical studies. Sections were incubated overnight at 4 °C with anti-E-cadherin antibody (BD Biosciences, North Brunswick Township, NJ, USA) and anti-ZEB1 antibody (Novus Biologicals, Centennial, CO, USA). Following this, the sections were treated with goat-anti-mouse IgG Alexa Fluor 488 and goat-anti-mouse IgG Alexa Fluor 555 for 1 h (both from Thermo Fisher Scientific, Waltham, MA, USA). The staining procedure was conducted using the Antifade Reagent with 4′,6-diamidino-2-phenylindole (DAPI) (Cell Signaling Technology, Danver, MA, USA), and cellular observation was performed under a BZ-X800 fluorescence microscope (Keyence Corporation, Osaka, Japan). This procedure was also followed for spheroids and organoids treated with Bevacizumab. Quantification of immunofluorescence intensity was performed using ImageJ software (Version 1.54k, National Institutes of Health, Bethesda, MD, USA). The integrated fluorescence intensity was measured by selecting regions of interest (ROIs) within each field. Background fluorescence was subtracted using adjacent non-stained areas within the same field. The fluorescence intensity values were normalized to DAPI nuclear staining intensity to account for variations in cell number and tissue thickness. Statistical analysis was performed using the Mann–Whitney U test to compare fluorescence intensities between spheroids and organoids. Data were expressed as mean ± standard deviation (SD) from five independent experiments. To ensure standardization across all samples, image acquisition parameters remained constant throughout the experimental series. All quantification procedures were performed on raw, unprocessed images to maintain data integrity.

### 2.9. Western Blotting

Total protein was extracted from spheroids and organoids using a lysis buffer supplemented with a protease/phosphatase inhibitor cocktail (both from Cell Signaling Technology, Danver, MA, USA). Protein concentrations were measured using a Nanodrop 2000 system (Thermo Fisher Scientific, Waltham, MA, USA). Proteins were separated by 10% sodium dodecyl sulfate–polyacrylamide gel electrophoresis (Bio-Rad Laboratories, Hercules, CA, USA) and transferred onto polyvinylidene difluoride membranes (Invitrogen, Carlsbad, CA, USA). Membranes were blocked with 3% skimmed milk for 1 h, then incubated with anti-E-cadherin antibody (BD Biosciences, San Jose, NJ, USA), anti-ZEB1 antibody (Novus Biologicals, Centennial, CO, USA), and anti-β-actin antibody (Cell Signaling Technology, Danver, MA, USA) at 4 °C overnight. Membranes were subsequently reacted with horseradish peroxidase (HRP)-linked secondary antibodies for 1 h at room temperature. Band intensities were quantified using an ImageQuant LAS 500 system (GE Healthcare, Chicago, IL, USA). The same experiments were repeated for spheroids and organoids treated with Bevacizumab or transfected with miR200c. Protein quantification was conducted using ImageJ (National Institutes of Health, Bethesda, MD, USA). For analysis, rectangular selections of equal size were applied to all bands. Background was subtracted using a rolling ball radius of 50 pixels. Band intensities were then normalized to the corresponding β-actin signal. Images were converted to 8-bit, and mean gray values were measured. Each experiment was performed in triplicate, and relative protein expression was calculated as the ratio of target protein to β-actin.

### 2.10. Statistical Analysis

Data representation and statistical analysis were conducted using the JMP 15.0.0 software program (SAS Institute, Cary, NC, USA). The Mann–Whitney U test was employed to assess differences between groups. Unless otherwise stated, data were presented as the mean ± standard error of the mean. Significance was considered at *p* < 0.05.

## 3. Results

### 3.1. In Vitro Development of EGFR-Mutant Lung Cancer Organoids

In an attempt to construct an innovative in vitro model, *EGFR*-mutant lung cancer organoids were established using HCC827 and H1975 cells, which are known to carry *EGFR* mutations. Notably, these cell lines were successful in forming complex, three-dimensional lung cancer organoids when co-cultured with MSCs and HUVECs.

**Figure 1:**
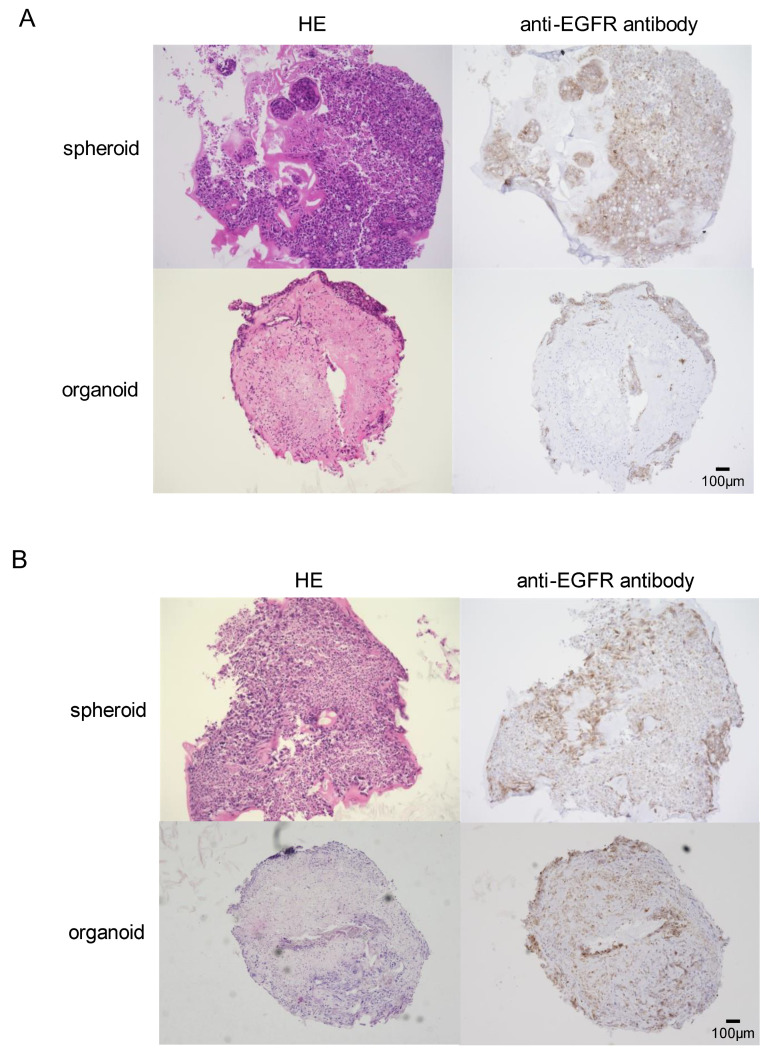
Construction and microscopic findings of spheroids and organoids using *Epidermal Growth Factor Receptor (EGFR)-*mutant lung cancer cells. (**A**) Hematoxylin and eosin (HE) staining and EGFR immunostaining of HCC827-derived spheroids and organoids. (**B**) HE and EGFR staining of structures generated from H1975 cells.

Detailed organoid formation was demonstrated in Figure 1, featuring immunohistochemical staining. Specifically, positive EGFR staining provided a distinct demarcation between the cancer cells and the stromal components, corroborating the successful incorporation of these cell types into the organoid structure. It was further noted that, compared to spheroids, the organoids showed a higher degree of stromal tissue composition, hinting at their superior potential in mimicking the natural tumoral microenvironment in vivo. Intriguingly, the morphological differences between the two *EGFR*-mutant lung cancer cell lines, HCC827 (Figure 1A) and H1975 (Figure 1B), remained insignificant within the organoid context. This consistent morphology suggests a potential underlying uniformity in the behavior of EGFR-mutant cells within organoids.

### 3.2. Differential Susceptibility of Spheroids and Organoids to EGFR-TKI

**Figure 2:**
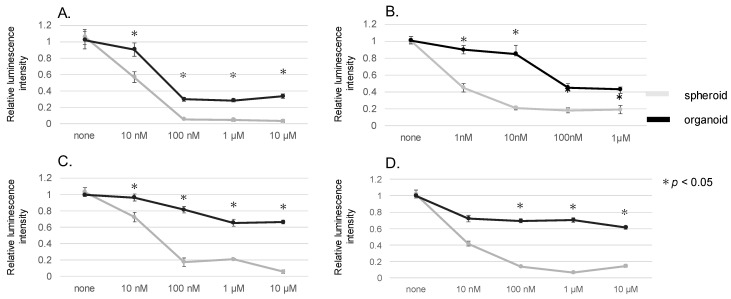
Assessment of cell viability in response to various EGFR-TKIs in spheroids and organoids derived from cancer cells, determined by a luciferase assay. Each panel displays the influence of an individual EGFR-TKI on spheroids or organoids: (**A**) Gefitinib on HCC827, (**B**) Afatinib on HCC827, (**C**) Osimertinib on HCC827, and (**D**) Osimertinib on H1975. Each experiment was repeated three times (n = 3), with * *p* < 0.05 (determined by the Mann–Whitney U test) indicating a statistically significant difference in cell viability.

To elucidate the differential response of spheroids and organoids to EGFR-TKI therapy, a series of experiments involving luciferase assays were conducted. The ensuing data, presented in Figure 2, unraveled marked differences in the therapeutic susceptibility of the two models.

In Figure 2A, the cell viability of HCC827 cells, subsequent to incubation with Gefitinib, is shown. Figure 2B,C depict the respective viability of HCC827 cells following treatment with Afatinib and Osimertinib. Figure 2D presents the results of the viability of H1975 cells post-treatment with Osimertinib.

A prominent finding from the experiments was the considerable elevation in IC50 values for all employed EGFR-TKIs in organoids in contrast to those in their spheroid equivalents (Figure 2A–D). This observation implies a heightened resistance to EGFR-TKI therapy within the organoid model.

### 3.3. Epithelial-to-Mesenchymal Transition in Organoids: Immunofluorescence Analysis of EMT Markers

**Figure 3:**
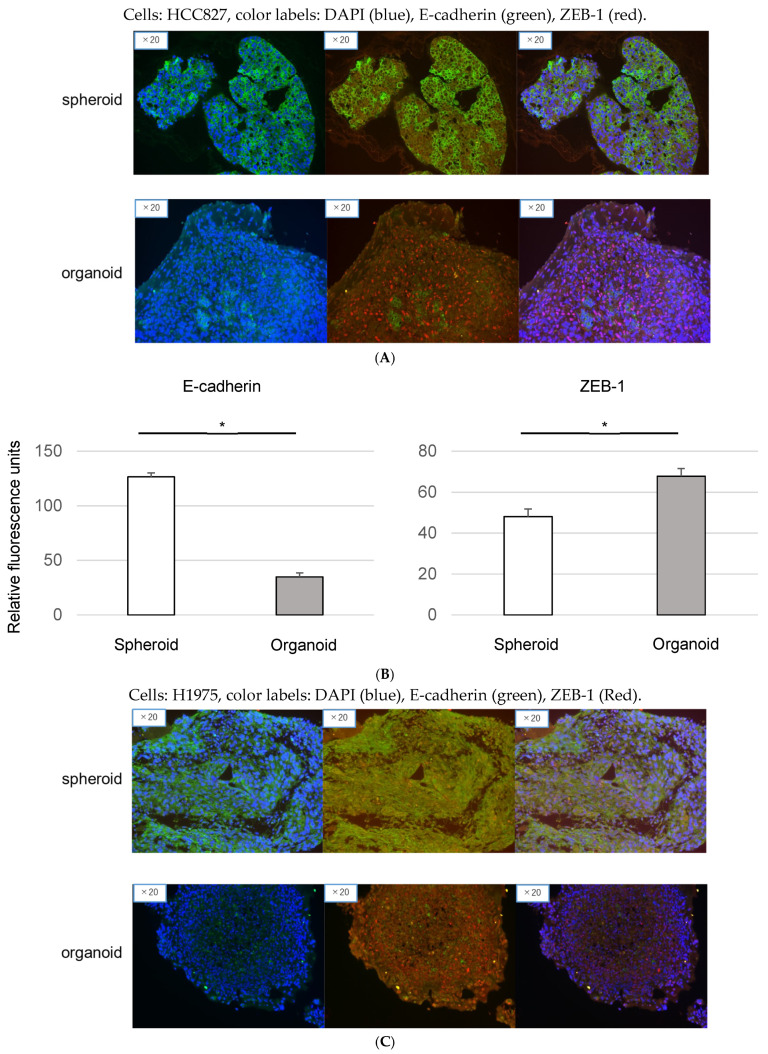

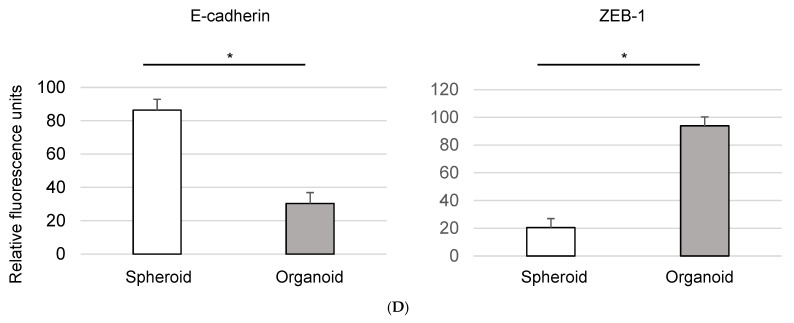
Immunofluorescence imaging of Epithelial Cadherin (E-cadherin) and zinc-finger-enhancer Binding Protein 1 (ZEB-1) in spheroids and organoids derived from lung cancer cell lines. (**A**) Visualization of these markers in HCC827-derived structures. The images illustrate cellular staining patterns for DAPI (blue, indicating cell nuclei), E-cadherin (green, an epithelial marker), and ZEB-1 (red, a mesenchymal marker). (**B**) Quantitative analysis of fluorescence intensity for e-cadherin and ZEB-1 in spheroids and organoids. the bar graphs depict the fluorescence intensity of E-cadherin and ZEB-1 in spheroids and organoids derived from *EGFR*-mutant lung cancer cells. Fluorescence intensity was measured and background-subtracted. Data represent the mean ± standard deviation (SD) of five independent experiments. Asterisks (*) indicate statistically significant differences between spheroids and organoids (*p* < 0.05, Mann–Whitney U test). (**C**,**D**) The same images for H1975-derived structures.

To substantiate the hypothesis of EMT-induced resistance to EGFR-TKI in organoids, immunofluorescence analyses were carried out to assess the expression of key EMT markers. The findings are visually represented in Figure 3, indicating significant variations between organoids and spheroids.

Figure 3A illustrates the upregulation of Epithelial Cadherin (E-cadherin, an epithelial marker) and the concurrent downregulation of zinc-finger-enhancer binding protein 1 (ZEB-1, a marker for EMT) in HCC827 organoids as compared to HCC827 spheroids. This expression pattern aligns with the characteristic signature of EMT, suggesting a more pronounced induction of EMT in organoids than in spheroids.

An analogous observation was made in the case of H1975 cells, as depicted in Figure 3B. The data confirmed a similar trend of EMT marker expression, reinforcing the suggestion that EMT may play a pivotal role in the observed EGFR-TKI resistance in organoids.

### 3.4. Modulation of EMT and EGFR-TKI Resistance in Organoids by Bevacizumab

The potential influence of Bevacizumab, an antibody against vascular endothelial growth factor (VEGF), on EMT and EGFR-TKI resistance in organoids is explored in the following section.

**Figure 4:**
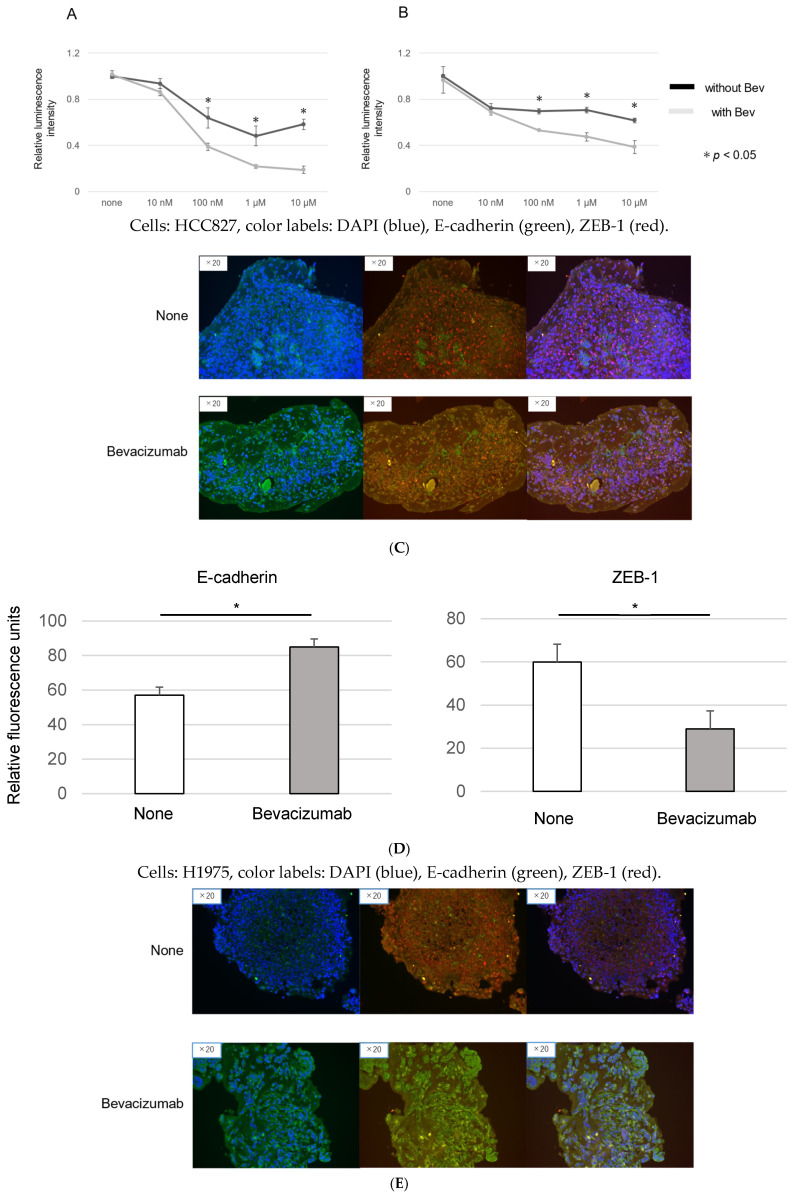

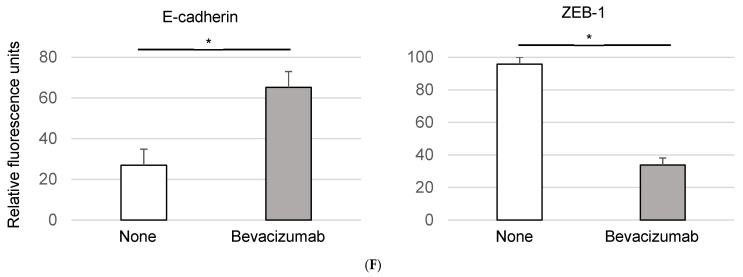
Analysis of the combined effects of Bevacizumab and EGFR-TKI on EGFR-mutant cancer cells. (**A**,**B**) The results of a luciferase assay measuring cell viability following Osimertinib treatment alone or in combination with Bevacizumab in HCC827 and H1975 organoids, respectively (n = 3, * *p* < 0.05, Mann–Whitney U test). (**C**) Immunofluorescence analysis of E-cadherin and ZEB-1 in HCC827. E-cadherin (green; left panels) and ZEB-1 (red; middle panels) expression and localization in HCC827 organoids, with and without Bevacizumab treatment. Merged images are shown in the right-hand panels. (**D**) Quantitative analysis of fluorescence intensity for E-cadherin and ZEB-1 expression in organoids. Values represent mean ± SD from three independent experiments (* *p* < 0.05, Mann–Whitney U test). (**E**,**F**) The corresponding analysis in H1975 organoids.

A luciferase assay was employed to discern the combined effects of Bevacizumab and EGFR-TKI on EGFR-mutant cancer cells. As demonstrated in Figure 4A for HCC827 cells and in Figure 4B for H1975 cells, the IC50 of the combined Osimertinib and Bevacizumab treatment was significantly lower than that of Osimertinib alone. These findings suggest an improvement in therapeutic efficacy, possibly linked to EMT suppression, with the addition of Bevacizumab.

Further examination of the EMT changes in response to Bevacizumab treatment was undertaken via immunofluorescence analyses in HCC827 organoids (Figure 4C,D). The results revealed an increase in E-cadherin-positive cells and a reduction in ZEB-1-positive cells, implying that the addition of Bevacizumab might attenuate EMT. A corresponding observation was made in the H1975 organoids (Figure 4E,F). Collectively, these results offer a promising direction for improving EGFR-TKI resistance in EGFR-mutant lung cancer. The observed EMT modulation by Bevacizumab highlights the potential utility of this VEGF inhibitor in conjunction with EGFR-TKIs for more effective treatment strategies.

### 3.5. Bevacizumab Inhibits on EMT in Cancer Organoids

**Figure 5:**
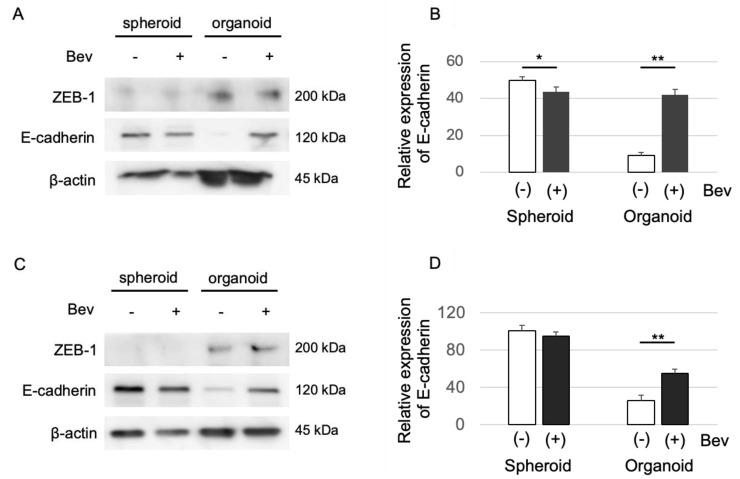
Western blot analysis examining E-cadherin and ZEB-1 expression in spheroids and organoids, with and without Bevacizumab treatment. (**A**) ZEB-1 (top) and E-cadherin (middle) levels in HCC827 spheroids and organoids. β-actin is utilized as a loading control (bottom panel). (**B**) Quantification of relative E-cadherin expression in HCC827 spheroids and organoids with or without Bevacizumab treatment. Data represent mean ± SD from three independent experiments. * *p* < 0.05, ** *p* < 0.001 (Student’s *t*-test). (**C**) Western blot analysis of ZEB-1, E-cadherin, and β-actin expression in H1975 spheroids and organoids treated with or without Bevacizumab. (**D**) Quantification of relative E-cadherin expression in H1975 spheroids and organoids with or without Bevacizumab treatment. Data represent the mean ± SD from three independent experiments. ** *p* < 0.001 (Student’s *t*-test).

The possible role of Bevacizumab in the inhibition of EMT within cancer organoids was scrutinized. The expression of E-cadherin and ZEB-1, critical markers of EMT, was assessed by Western blotting following the culture of HCC827 cells (Figure 5A,B) and H1975 cells (Figure 5C,D) with Bevacizumab.

The data presented in Figure 5 reveal that E-cadherin expression was enhanced in organoids treated with Bevacizumab, in contrast to that in those without the treatment. This upregulation of the epithelial marker E-cadherin is indicative of a suppression in EMT, thereby suggesting an ameliorative effect of Bevacizumab on the EMT process.

### 3.6. miR200c Modulates EMT and Enhances EGFR-TKI Sensitivity in Organoids

**Figure 6:**
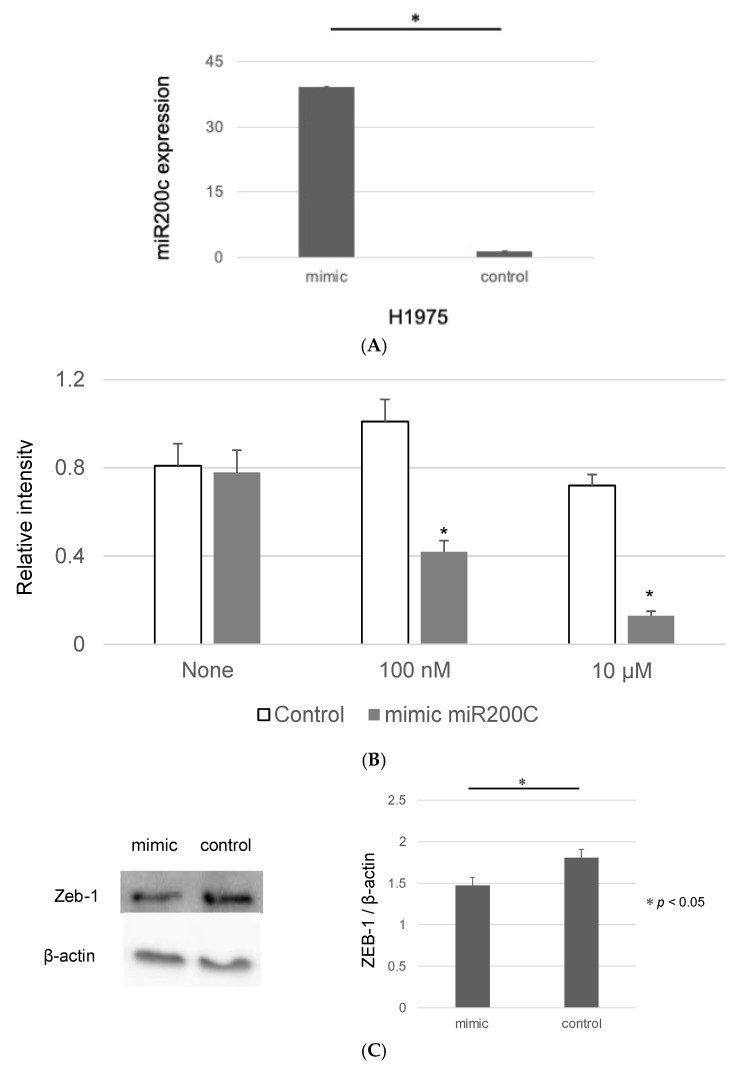
The effects of miR200c on *EGFR*-mutant lung cancer organoids. (**A**) RT-PCR analysis comparing the expression of miR200c between organoids transfected with miR200c mimic and control organoids (n = 3, * *p* < 0.05, Mann–Whitney U test). (**B**) Luciferase assay depicting cell viability in organoids treated with Osimertinib with or without the addition of the miR200c mimic (n = 3, * *p* < 0.05, Mann–Whitney U test). (**C**) Western blot analysis in spheroids and organoids with or without the introduction of the miR200c mimic, assessing the expression of ZEB-1 and β-actin. The bar graph, created using Image J, compares protein quantifications (n = 3, * *p* < 0.05, Mann–Whitney U test).

As previous reports have suggested that miR200c reduces EMT in cancer cells through the regulation of ZEB-1 [19], we aimed to investigate the role of miR200c in modulating the sensitivity of organoids to EGFR-TKIs. To this end, a mimic of miR200c was transfected into the organoids, and a significant increase in miR200c levels was observed in H1975 organoids compared to controls (Figure 6A).

Following this, the impact of the miR200c mimic on the susceptibility of organoids to EGFR-TKIs was assessed via a luciferase assay. The IC50 of organoids transfected with the miR200c mimic was significantly lower than that of the controls, suggesting enhanced drug sensitivity (Figure 6B).

Regarding the expression level of ZEB-1, a known target of miR200c and an EMT marker, we observed a significant decrease in ZEB-1 expression in H1975 organoids after transfection with the miR200c mimic, compared to the controls (Figure 6C).

Transfection of mimic miR200c was achieved successfully in H1975 spheroids and organoids; however, similar attempts in HCC827-derived models were unsuccessful despite multiple optimization attempts. This observation suggests cell-line-specific variations in miRNA uptake or processing.

## 4. Discussion

We established spheroids and organoids by culturing lung cancer cells with EGFR mutation, MSCs and HUVECs on matrigel. The results of cell viability analysis indicated that the organoids had more drug resistance compared to the spheroids. An increase in ZEB-1 and a decrease in E-cadherin were shown in the organoids, which indicated that the drug resistance might have been caused by EMT. Adding Bevacizumab as well as miR200c to the organoids resulted in the improvement of drug resistance and EMT.

EGFR-TKIs have significantly improved treatment for NSCLC patients with activating EGFR mutations. However, TKI resistance often develops, leading to disease progression, typically within a couple of years. Therefore, finding new drugs or strategies to counteract TKI resistance and enhance patient outcomes in advanced NSCLC is a priority [20,21].

EMT and its role in drug resistance are emerging areas of interest in cancer research. Numerous EMT-related signaling pathways have been implicated in cancer cell drug resistance [22]. Furthermore, EMT is one of the key resistance mechanisms against EGFR-TKIs including other mechanisms such as the ’gatekeeper’ T790M mutation, histologic transformation from NSCLC to small-cell lung cancer, and concurrent molecular or genetic alterations [23]. Additional mechanisms include the activation of alternative signaling pathways like *MET* amplification and HER2 overexpression [24,25]. Research has capitalized on in vitro models to explore mechanisms of resistance to anticancer drugs, specifically those linked to EMT. These models offer a controlled environment for understanding the intricacies of drug resistance and for trialing novel therapeutic approaches. Liu et al. investigated the association between cancer cell stemness and EMT characteristics within drug-resistant esophageal cancer cells [26].

In this, we utilized organoid models comprising MSCs and HUVECs. This facilitated the examination of in vitro interactions between diverse cellular entities within the TME [27]. Notably, when MSCs are cultured alongside cancer cells, they can potentially give rise to cancer-associated fibroblasts (CAFs) [28]. These CAFs are critical components of the TME. Our organoids demonstrated a significant presence of stromal tissues (Figure 1), possibly representative of CAFs within the TME. Interestingly, the phenomenon of EMT can be stimulated by CAFs within tumor cells [29]. One important marker of EMT is the downregulation of E-cadherin, a protein critical for cell–cell adhesion within epithelial tissues. Notably, decreased E-cadherin levels have been correlated with EMT and poor prognoses in lung cancer patients [30,31]. Additionally, ZEB 1, a transcription factor, is known to be inversely correlated with E-cadherin expression, further underlining its role in EMT [18]. In line with these findings, our study revealed decreased E-cadherin and increased ZEB 1 expressions within the organoids (Figure 2 and Figure 3). This shift in protein expression potentially indicates the induction of EMT and resultant drug resistance, underscoring the relevance of our model in studying these critical processes in NSCLC.

Bevacizumab, an anti-angiogenic agent, functions by inhibiting VEGF signaling pathways. Its dual effects involve impeding angiogenesis to deprive the tumor of oxygen and normalizing the vasculature to enhance treatment sensitivity [32]. The addition of Bevacizumab to Erlotinib has been observed to extend progression-free survival in NSCLC patients compared to Erlotinib monotherapy [33]. In our investigation, Bevacizumab appeared to ameliorate drug resistance and EMT (Figure 4, Figure 5 and Figure 6). There is evidence to suggest that elevated VEGF expression in malignant cells may drive EMT [34]. The existing literature presents conflicting findings about the relationship between Bevacizumab and EMT. For instance, Kim et al. demonstrated that Bevacizumab could inhibit TGF-β1-induced EMT in colon cancer cells [35]. However, in stark contrast, another study reported that Bevacizumab promoted EMT through Wnt/β-catenin signaling in glioblastoma cells. Further research should focus on clarifying the role of VEGF and the effects of Bevacizumab [36].

We also explored the role of miR200c, in relation to EMT in cancer organoids. Prior research has shown that miR200c plays a pivotal role in EMT among several cancers [37]. In EGFR-mutant lung cancer cell lines displaying EMT features, a downregulation of miR200c was observed, attributable to the increased level of ZEB-1 in these cells [38]. ZEB-1, an EMT-promoting transcription factor, appears to be regulated by miR200c. Upon transfecting organoids, which exhibited a more pronounced EMT than cell lines, with miR200c, we noticed an improvement in EMT and drug resistance, accompanied by a decrease in ZEB-1 (Figure 6). However, attempts to transfect miR200c into HCC827 organoids were unsuccessful, likely due to cell-line-specific differences in miRNA uptake or intracellular processing mechanisms. This limitation underscores the variability in cellular responses across different EGFR-mutant lung cancer models and highlights the need for alternative transfection strategies or delivery systems to fully elucidate miR200c’s role in these contexts.

Despite this, our findings reinforce the potential of miR200c as a promising therapeutic agent for targeting EMT and overcoming chemotherapy resistance. Future studies should address these technical challenges and further investigate miR200c’s efficacy in broader EGFR-mutant contexts.

Our study is not without limitations. Firstly, the TME replicated in our organoids does not fully encapsulate the intricate characteristics of the TME in human cancers. Elements such as immune cells, exosomes, and vessels, which play a crucial role in the human cancer milieu, are absent in the organoids. One of the limitations of this study is the difficulty in accurately assessing cancer cell-specific viability within organoid models, as these organoids consist of multiple cell types, including mesenchymal stem cells and endothelial cells. Thus, there is an imperative need to validate these results in in vivo models. Secondly, it should be noted that our study specifically focused on EGFR-mutant lung cancer cells (del 19 or L858R/T790M) and their EMT-mediated resistance to EGFR-TKIs. While these mutations are clinically relevant targets for EGFR-TKI therapy, the interaction between EGFR mutations and EMT development may vary among different mutation types. Clinical trials have shown varying efficacies of EGFR-TKIs across different EGFR mutation patterns, suggesting that the EMT process and its reversal by Bevacizumab or miR200c might also show mutation-specific responses. Therefore, our findings should be interpreted within the context of these specific EGFR mutations, and further studies may be needed to validate these results across different EGFR mutation variants.

Despite the several novel findings, our study is not without limitations. Firstly, the TME replicated in our organoids does not fully encapsulate the intricate characteristics of the TME in human cancers. Elements such as immune cells, exosomes, and vessels, which play a crucial role in the human cancer milieu, are absent in the organoids.

Thus, there is an imperative need to validate these results in in vivo models.

Secondly, the lung cancer cells employed in this study harbor specific EGFR mutations (del 19 or L858R/T790M), which could potentially bias the results. Thirdly, while miR200c transfection was successful in H1975 cells, similar attempts in HCC827 cells were unsuccessful despite multiple optimization efforts, suggesting potential cell-line-specific variations in miRNA processing or uptake mechanisms that warrant further investigation.

## 5. Conclusions

Our study provided compelling evidence suggesting that EMT is a key mechanism of resistance against EGFR-TKIs, emphasizing the necessity of EMT-targeting strategies in overcoming this resistance. Our in vitro organoid models offered valuable insights into the tumor microenvironment, while the role of Bevacizumab and miR200c in modulating EMT was further highlighted. Future research should aim to elucidate these interactions more precisely and explore their potential in clinical applications.

## Data Availability

The data presented in this study are available on request from the corresponding author.
